# Overcoming Challenges in Avian Influenza Diagnosis: The Role of Surface-Enhanced Raman Spectroscopy in Poultry Health Monitoring

**DOI:** 10.3390/vetsci12111052

**Published:** 2025-11-02

**Authors:** Muhammad Farhan Qadir, Yukun Yang

**Affiliations:** 1 College of Life Sciences, Henan Normal University, Xinxiang 453007, China; 2 School of Life Science, Shanxi University, Taiyuan 030006, China

**Keywords:** avian influenza, virus, surface-enhanced Raman spectroscopy, SERS, public health, challenges, chicken, detection methods

## Abstract

**Simple Summary:**

Outbreaks of avian influenza (AI/bird flu/avian flu) cause severe economic damage to the poultry sector worldwide, primarily through the mandatory culling of infected flocks. Several AI variants demonstrate zoonotic potential, capable of infecting a broad host range that includes humans, birds, and other animals, thereby posing a significant global pandemic threat. Rapid and precise methods are essential for effective surveillance and early intervention to mitigate outbreak potential. Surface-enhanced Raman Spectroscopy (SERS) is a highly sensitive method applied in scientific investigations to identify and examine even small amounts of substances. This method has been modified to identify viruses such as bird flu and many others. The fundamental concept of SERS involves illuminating a sample with a laser beam and examining the reflected light. Each molecule exhibits a unique Raman spectral fingerprint due to its distinct interaction with light, enabling precise sample characterization. However, a primary challenge in direct viral detection is the characteristically low concentration of virions in clinical samples, which often yields a signal below the detection limit of conventional Raman spectroscopy. So, SERS addresses the critical challenge of low viral concentration by employing gold or silver nanoparticles to significantly amplify the Raman signal, thereby enabling sensitive virus detection. This review explores the limitations of conventional Avian Influenza (AI) diagnostics and evaluates SERS-based applications as rapid alternatives. This review critically evaluates traditional and novel detection platforms, performs a comparative analysis of SERS against established viral testing methods, and proposes novel AI prevention strategies.

**Abstract:**

Rapid and accurate diagnostics for influenza viruses are essential for preventing future epidemics. Surface-enhanced Raman spectroscopy (SERS) presents a promising alternative to conventional techniques, offering a rapid, cost-effective, and highly sensitive platform for influenza virus detection. It is a highly sensitive analytical technique that enables the detection of minute chemical substances through significant signal enhancement. It operates by illuminating a sample with a laser and analyzing the scattered light to generate a unique molecular Raman spectrum. The sensitivity of SERS is derived from its use of metal nanoparticles, which amplify the weak Raman signals, making it particularly effective for detecting low-concentration targets such as viruses. Avian influenza (AI) is a major threat to domestic poultry, leading to large-scale culling during outbreaks. It leads to economic losses globally and can also infect pigs and humans, potentially causing a pandemic. Migratory birds spread various strains, leading to the development of highly pathogenic viruses. Viral monitoring is crucial for prevention strategies and understanding the virus evolution. This review outlines the challenges in detecting AI virus in chickens and critically assesses the established and emerging diagnostic technologies, with a specific focus on the factors influencing detection and recent advances in SERS-based AI detection. Ultimately, this review aims to provide insights that will assist the influenza research community in developing novel strategies for monitoring and preventing AI outbreaks in chickens and mitigating zoonotic transmission.

## 1. Introduction

**Figure 1:**
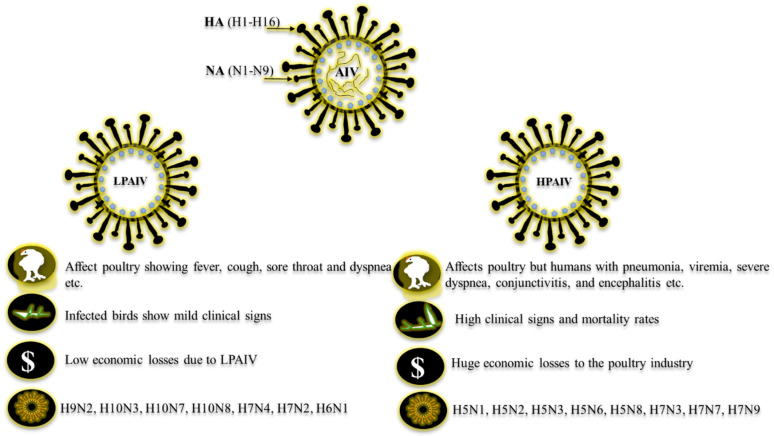
Morphological structure of AIV and comparison between HPAIV and LPAIV with their respective strains. Data from [2,3,4,5,6,8,67].

Several pathogens cause major economic losses in the poultry industry [1,2,3,4,5,6]. One key group is the influenza viruses, segmented RNA viruses, belonging to the Orthomyxoviridae family [7]. These viruses are categorized into four genera (A, B, C, and D), and the avian influenza virus (AIV) is a subtype of influenza A. Among them, birds are affected by only Influenza A viruses, and these viruses are subtyped according to their hemagglutinin (H) and neuraminidase (N) glycoproteins. AIVs are classified into 16 hemagglutinin (H1–H16) and 9 neuraminidase subtypes (N1–N9) [2] (Figure 1). Both proteins divide and distinguish different AIV serotypes by analyzing their genetic differences [2,7,8]. A recent study has also identified 19 (H1–H19) hemagglutinin and 11 (N1–N11) neuraminidase subtypes [3]. AIVs are categorized into two pathotypes based on their ability to induce the disease: highly pathogenic AIV (HPAIV) and low-pathogenic AIV (LPAIV) [2,4,5,6], and related strains are depicted in Figure 1. Avian pathogenesis starts with the inhalation or ingestion of a pathogenic influenza strain (LPAIV or HPAIV). Subsequent systemic dissemination can result in infection of vital organs, potentially leading to multi-organ failure, severe clinical manifestations, and mortality [9,10,11].

**Figure 2:**
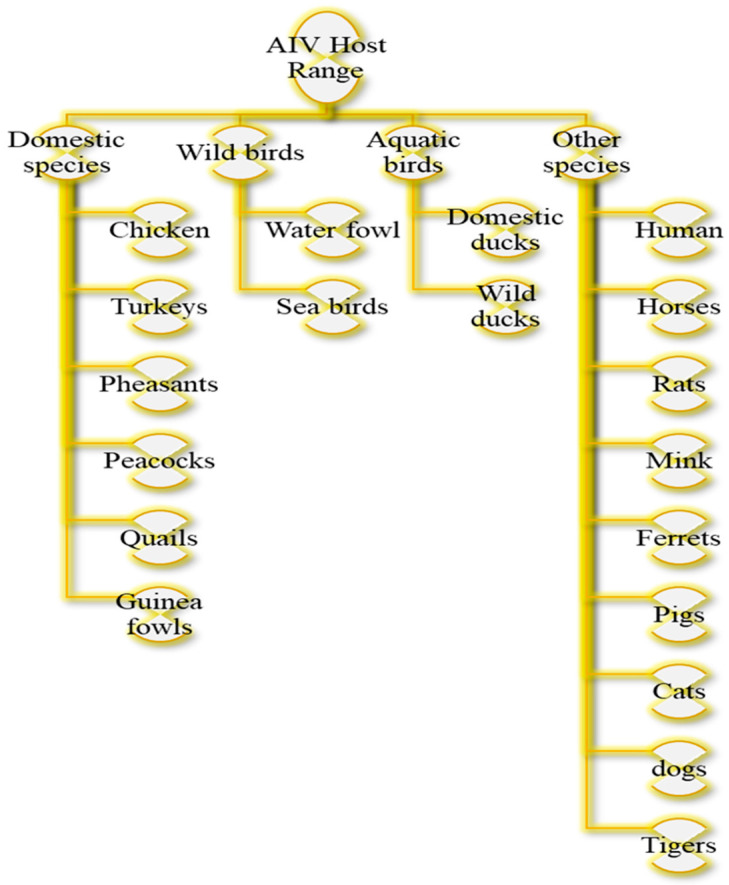
Classification of AIV host range. Data from [10,11,15,16,17].

**Figure 3:**
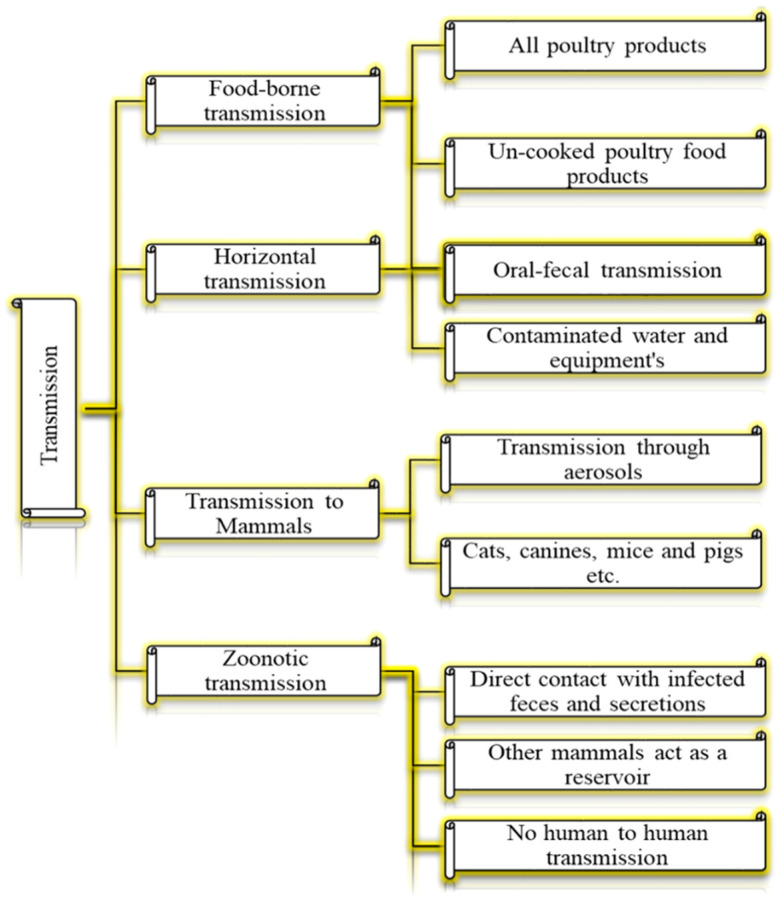
Transmission routes of AIV. Data from [17,18,20,22,26,27,30,32,34,36].

Birds infected with HPAIV or LPAIV can manifest in a wide spectrum of clinical signs and pathological lesions in birds. Severe cases can result in mortality [9,12,13,14]. Infected birds can transmit the virus for up to 200 days. The virus can persist in dead bodies, eggs, and meat, necessitating proper disposal [11,15,16]. AI can impact various bird species, domestic poultry, humans, rodents, and pets (Figure 2). The primary hosts during outbreaks are domestic poultry, while wild birds often serve as carriers [10,11,17]. AIV is primarily maintained in migratory waterfowl and spreads to domestic poultry via the oral–fecal route through contaminated environments. Infected birds can transmit the virus by shedding it in their feces for up to 21 days [18,19]. The virus is transmitted through different routes [20,21,22,23,24,25,26,27,28,29,30,31,32,33,34,35,36] (Figure 3).

Current methods for detecting respiratory viruses, including virus isolation [37,38] and culture, polymerase chain reaction (PCR) [39], and enzyme-linked immunosorbent assay (ELISA) [40,41], are highly sensitive. However, they require rigorous conditions, are time-consuming, and depend on sophisticated instrumentation and skilled personnel. Several alternative methods have been developed to detect AIV, including quartz-crystal microbalance (QCM) [42], electrochemical methods [43], surface plasmon resonance (SPR) [44], and fluorescent and colorimetric immunosensors [45,46,47,48]. However, many of these biosensors remain impractical for widespread use due to time-consuming procedures, insufficient sensitivity, and a lack of portability [49].

Existing techniques still have limitations/potential for enhancement in the usability, sensitivity, and practicality. Consequently, a sensitive, specific, and rapid detection method such as SERS is essential for the control of the pandemic influenza viruses. This technology enables early diagnosis, facilitates the start of antiviral treatment, and provides essential surveillance, especially for high-risk populations [4]. SERS is a powerful analytical technique, discovered in the mid-1970s, that significantly enhances the Raman scattering signal of molecules adsorbed on or near specially prepared nanostructured metal surfaces, typically made of gold or silver. This dramatic amplification effect allows for the highly sensitive detection and identification of chemical compounds and biological materials, even at the single-molecule level [50,51,52,53,54]. Scientists are exploring SERS, focusing on chemical enhancements, electromagnetic and single-molecule detection, and how substrate structure affects optical response [55,56,57,58]. SERS has diverse applications in identification and detection. It is used to identify biological organisms, food additives, and contaminants; detect explosives; assist in forensic investigations; and monitor reactions involving nanoparticles or metallic surfaces [59,60,61,62,63]. As a result, SERS has been widely applied across various fields for the precise analysis of a diverse range of targets, including small molecules, biomarkers [64], proteins, nucleic acids, and viruses [40,65,66].

The need for rapid, on-site AIV detection in chickens and live bird markets is critical for outbreak prevention and control. Supplementary Table S1 provides a comparative analysis of SERS against traditional methods (RT-PCR, ELISA, LAMP, etc.), summarizing key advantages, disadvantages, and current technological gaps. Despite its promising advantages, SERS must overcome several gaps to become a mainstream technique for AIV. A critical barrier to the implementation of SERS technology is the lack of large-scale, comparative field validation. Future research must prioritize collaborative studies with veterinary diagnostic networks to benchmark SERS against gold-standard methods like qPCR, ELISA, and LAMP using hundreds of field samples, thereby providing a definitive assessment of its cost-effectiveness, diagnostic accuracy, and operational practicality.

This review analyzes the current challenges, the key factors influencing SERS-based diagnosis, and the applications of SERS-based AIV detection in chickens. Moreover, a newly developed SERS-based method, reviewed herein using collated data from multiple databases, provides a precise, convenient, and simpler on-site detection of AIV.

## 2. Detection Methods

The efficacy of AIV detection is fundamentally determined by the surveillance strategy. Passive surveillance, dependent on reporting clinical signs, is effective for identifying HPAIV outbreaks characterized by high mortality. In contrast, LPAIV strains, which often cause mild or asymptomatic infections, frequently evade this method. Consequently, active surveillance involving systematic farm visits and diagnostic testing is crucial for comprehensive AIV monitoring. Furthermore, the clinical manifestation of AI is highly variable and is influenced by factors including viral subtype, host species, age, and immune status [16].

The identification and isolation of AIV remain crucial, relying on the cultivation and characterization of distinct subtypes to determine the outbreak potential. Since the development of the golden method, i.e., the use of embryonated chicken eggs for AIV propagation, a technique developed in the 1930s [68], researchers have consistently reported adaptations in influenza viruses resulting from this method [69]. However, the traditional method of virus isolation has limitations for rapid response, especially for HPAIV, which requires the stringent safety protocols of a BSL-3 lab [11,70]. Serological assays, particularly various ELISA techniques, serve as a practical and cost-effective tool for AIV surveillance by detecting antiviral antibodies in poultry. However, despite their widespread use, these tests can be limited by drawbacks such as cross-reactivity with other antigens, variable sensitivity, and varying reproducibility, especially in specific antigen detection [71,72,73,74,75,76,77,78,79,80,81,82,83,84,85].

Immunological techniques, such as colloidal gold immuno-chromatography and fluorescence immunochromatography, use different labeling agents to detect viruses. Colloidal gold immuno-chromatography uses colloidal gold to create an antigen–antibody complex visible to the naked eye [86], while fluorescence immunochromatography [87,88] utilizes fluorescent nanomaterials like quantum dots for improved sensitivity and specificity in virus detection. Quantum dots offer optical benefits such as strong fluorescence and customized emission properties, making them valuable tools in virus detection technologies [67,89,90].

Reverse transcription polymerase chain reaction (RT-PCR) is a crucial technique for amplifying specific DNA fragments in various fields, like microbial detection and aquaculture [91]. It involves creating cDNA from mRNA using reverse transcriptase [92], followed by amplification [93]. Recombinase Polymerase Amplification (RPA) [67] relies on recombinase to merge single DNA strands during amplification, offering benefits such as easy operation and rapid detection. Various diagnostic tools, including Loop-Mediated Isothermal Amplification (LAMP) [94,95,96,97,98,99], Nuclear Acid Sequence-Based Amplification (NASBA) [100,101,102,103,104,105,106,107], gene chip technology [108,109,110,111,112,113], Next-Generation Sequencing (NGS) [114,115], and biosensors [44,116,117,118,119,120,121,122,123,124,125,126,127,128,129,130,131,132,133,134,135,136,137], have the potential to provide cost-effective pathogen detection with their accuracy, specificity, and selectivity, offering a promising solution for easy, quick, and cost-effective detection.

Accurate and early detection of AIVs is essential for managing outbreaks and preventing severe, often fatal, respiratory illness in humans. Conventional techniques, however, are hampered by significant limitations in their sensitivity, specificity, and reaction time. SERS offers a powerful alternative for AIV detection. Primarily, its enhancement processes provide superior sensitivity over traditional Raman spectroscopy, allowing for the detection of minimal viral concentrations [138]. This technique also delivers a unique molecular fingerprint of AIVs, allowing for specific identification and differentiation of different viral strains, with the potential for multiplexing, enabling the simultaneous detection of multiple viral targets [15]. As a rapid, label-free, and non-destructive technique, it requires minimal sample preparation and provides results within minutes without damaging the sample [139,140,141]. These features, including its adaptability to portable devices for point-of-care use, make SERS a highly promising tool for efficient and effective AI surveillance and management in poultry [139,142,143,144,145,146]. The potential of SERS for AIV detection is well-demonstrated by several key studies. Chaudhary et al. [147] successfully differentiated AIV strains H5N2 and H7N2 using silver nanorod arrays, achieving a detection limit of 10^3^ particles/mL and attributing the distinctive SERS spectra to viral proteins and nucleic acids. In another study, Shanmukh et al. [148] developed a method for H5N1 using silver-coated silica nanorods, detecting the virus in allantoic fluid at 10^4^ particles/mL and confirming specificity against other influenza subtypes. These findings underscore the technique’s capacity for rapid, sensitive, and specific detection, which is vital for early diagnosis and outbreak management. However, to fully realize this potential requires further optimization of substrates, development of standardized protocols, and rigorous validation with clinical samples [148,149].

The optimal diagnostic method depends on the specific application requirements, including the sensitivity, turnaround time, available resources, and required throughput. For maximum sensitivity, RT-qPCR remains the established gold standard, while SERS emerges as a powerful alternative with the additional benefits of rapid analysis and intrinsic multiplexing capability. In point-of-care settings demanding portability and speed, both SERS and LAMP are suitable; SERS offers superior multiplexing, whereas LAMP provides a simpler, nucleic acid-based approach. For large-scale screening programs where cost-effectiveness and high throughput are priorities, ELISA remains the preferred choice, despite its lower sensitivity. Finally, when rapid, laboratory-quality results are needed at the point-of-care, SERS provides significantly higher sensitivity than conventional rapid antigen tests [150], although at a higher cost (Supplementary Table S1).

In conclusion, the application of SERS in detecting AIV in chickens has shown significant advancements through innovative studies and approaches. The diverse applications of SERS, ranging from rapid antigen tests to sound analysis, highlight the versatility and potential of this technology in poultry health monitoring. The integration of SERS with portable devices offers the prospect of real-time and on-site AIV surveillance and empowers more effective control and prevention strategies in the poultry industry.

## 3. Public Health Importance of AIV

AI is a major global public health concern due to its zoonotic capacity. Furthermore, the virus’s expanding host range is evidenced by increasing reports of outbreaks in wild and captive mammals [35,151,152,153]. The H5N1 subtype, known as the most well-known zoonotic AIV, was initially identified in humans in Hong Kong in 1997 and later resurfaced in mainland China in 2003 [154]. There have been reports of human cases worldwide involving different subtypes like H5N1 (870 cases and 430 deaths), H5N6 (80 cases and 30 deaths), H9N2 (80 cases and 2 deaths), H7N9 (1500 cases and 600 deaths), and sporadic cases of H3N8, H7N4, H7N7, and H10N3 [153,155]. From 2003 to 2022, 868 individuals were infected with influenza A (H5N1), and 457 of them died in 21 countries, according to reports to the Pan American Health Organization (PAHO)/WHO. In 2022, there have been multiple human cases of AI reported: the UK, Canada, and Spain observed cases of HPAIV (H5N1) in poultry farmers, while Asian countries have documented cases of infections caused by various subtypes such as H3N8, H5N6, and H9N2, including one reported fatality [16,151].

## 4. Factors Influencing the SERS-AIV Detection

SERS has emerged as a powerful tool for detecting Avian Influenza Virus (AIV) in poultry, offering enhanced sensitivity and specificity. Several key factors influence its practical application; First, how samples are collected, processed, and prepared for SERS analysis can significantly impact the detection sensitivity and accuracy. Proper sample preparation techniques are essential to ensure that the target viral particles are effectively captured and concentrated for analysis [156,157]. The choice of substrate used in SERS analysis can affect the enhancement of Raman signals from the target molecules. The sensitivity and specificity of SERS detection are directly governed by the optimization of substrate properties, including composition, morphology, and surface chemistry [158,159]. The optimization of laser wavelength and power is critical in SERS-based AIV detection, as these parameters directly influence the excitation efficiency of Raman signals from viral particles. Proper tuning is essential to maximize the signal-to-noise ratio and achieve high detection sensitivity [160,161]. To optimize SERS for AIV detection, it is crucial to understand the underlying signal enhancement mechanisms: electromagnetic, chemical, and charge-transfer. So, optimizing these enhancement mechanisms leads to more efficient and reliable detection [162,163]. The presence of complex biological matrices in chicken samples, such as proteins, lipids, and other biomolecules, can interfere with the SERS detection. Minimizing matrix effects through proper sample preparation and data analysis techniques is essential for accurate virus detection [164,165]. Factors such as temperature, humidity, and ambient light can impact the stability and reproducibility of SERS measurements. Controlling these environmental conditions during sample analysis is critical for obtaining reliable and consistent results in detecting AIV in chickens [166].

The viral load and shedding patterns of the AIV play a critical role in the detection sensitivity of SERS. Higher viral loads may result in stronger Raman signals, enhancing the detectability of the virus in infected chickens [167]. The level of biosecurity on poultry farms directly influences AIV occurrence. Consequently, rigorous measures that reduce viral transmission may also lower viral loads to levels that challenge SERS detection sensitivity [168,169,170]. Variations in farming practices, such as flock density, hygiene protocols, and contact with wild birds, can affect the likelihood of AIV introduction and circulation on chicken farms. These factors may influence the efficacy of SERS detection in AI-infected chickens [168]. The diversity of AIV strains circulating in poultry populations can impact the performance of SERS detection. Different strains may exhibit varying antigenic properties and shedding patterns, which influence the sensitivity and specificity of SERS in detecting specific viral strains [171,172]. The effectiveness of SERS-based AIV surveillance is contingent upon proper sample collection and handling, which are directly influenced by farmer awareness of AI control measures and compliance with farm-level biosecurity protocols [170]. The optimization of these parameters is crucial for maximizing the efficacy of SERS-based AIV detection, which in turn strengthens overall disease surveillance and control strategies in poultry.

### 4.1. Enhancement Mechanisms in SERS

The improvement in SERS is ascribed to two primary mechanisms: electromagnetic enhancement and chemical enhancement.

#### 4.1.1. Electromagnetic Enhancement

Electromagnetic enhancement is the dominant mechanism in SERS, contributing to an enhancement factor of up to 10^8^ [55]. When the incident laser light interacts with the metal surface, it excites the conduction electrons, creating localized surface plasmons. These surface plasmons create an enhanced electromagnetic field at the metal surface, which can interact with the analyte molecules. The magnitude of the electromagnetic enhancement depends on the size, shape, and composition of the metal nanostructures, as well as the wavelength of the incident light [173].

#### 4.1.2. Chemical Enhancement

Chemical enhancement, which contributes to an enhancement factor of up to 10^2^, is caused by the formation of a charge-transfer complex between the analyte and the metal surface. This charge-transfer complex alters the polarizability of the analyte, leading to an increase in the Raman scattering cross-section [174].

### 4.2. Sensitivity and Specificity of SERS in Detecting AIV Strains

The persistent threat of AI represents a significant and ongoing threat to both veterinary and public health, underscoring the critical need for the development of advanced diagnostic technologies. RT-PCR offers a highly sensitive approach for detecting various strains of Influenza Virus A and B, including the H5N1 subtype. This method enables the rapid and reliable detection of Influenza A and B viruses, providing a valuable tool for early diagnosis and outbreak intervention. Moreover, the sensitivity of SERS in detecting AIV strains is underscored by its ability to enhance the Raman signal of viral particles adsorbed on nanostructured metal surfaces. The high sensitivity of SERS makes it a promising technology for the rapid and accurate identification of AIV strains [138]. In addition, SERS offers exceptional specificity in detecting AI virus strains. The molecular analysis demonstrates specific identification of Influenza A virus H5 subtypes, highlighting the precision of SERS in differentiating between viral strains. The SERS-based detection assay demonstrated excellent diagnostic performance, validating high sensitivity and specificity across multiple Influenza A and B subtypes while showing no cross-reactivity with other common respiratory pathogens. The specificity of SERS in AIV detection is crucial for the accurate identification and differentiation of viral strains, especially in outbreak scenarios. The unique molecular fingerprints provided by SERS enable the highly specific and confident detection of Influenza A virus H5 subtypes [138,175].

## 5. SERS Applications for AIV Detection

The representative data related to SERS-based detection of AIV are summarized in Supplementary Table S2.

### 5.1. SERS-Lateral Flow Immunoassay-Based AI Viral Detection

**Figure 4:**
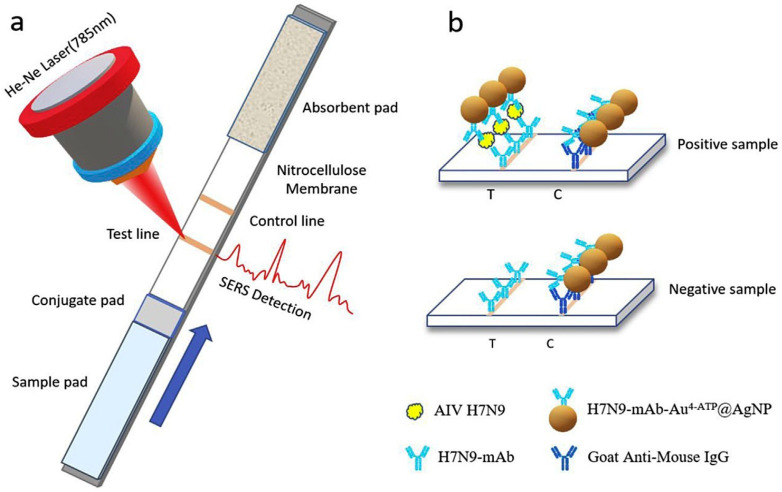
(**a**) SERS-LFIAS-based H7N9 AIV detection. (**b**) In the case of a positive sample, two brown lines were observed on the test line and control line; In the case of a negative sample, only one line appeared on the control line. Copyright/license 6100551025566; reproduced with permission from [176].

**Figure 5:**
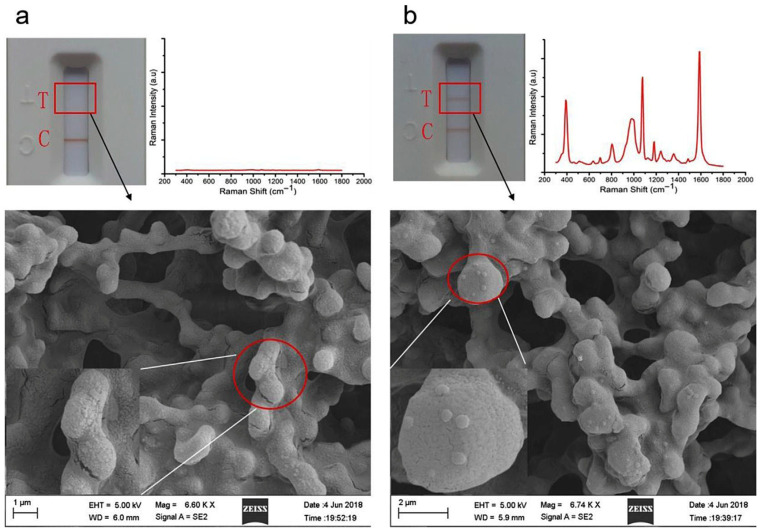
Photographic image, SERS spectra, and SEM images of the SERS-LFIAS: (**a**) in the absence of H7N9 AIV and (**b**) in the presence of H7N9 AIV (0.5 HAU). Copyright/license 6100551025566; reproduced with permission from [176].

Xiao et al. [176] investigated a novel method that integrates SERS with a lateral flow immunoassay strip (LFIAS) for the rapid and precise identification of AIV H7N9. The presence of H7N9 AIV was confirmed by the color change in the test line, and the concentrations of H7N9 AIV were quantified by averaging the SERS signals. The LFIA strip employed a unique core–shell structured material called AuAg^4−ATP^@AgNPs, serving as a Raman probe. An antibody that targets AIV and a goat anti-mouse IgG antibody were affixed to a nitrocellulose membrane as the test and control lines, respectively (Figure 4). Additionally, the LOD for the SERS-based LFIAS in detecting AIV H7N9 was found to be 0.0018 HAU (hemagglutination units). SEM images confirmed the capture of mAb-AuAg4-ATP@AgNPs on the test line for the H7N9 virus. Without AIV H7N9, no immunocomplexes formed (Figure 5a). While with 0.5 HAU AIV H7N9, immunocomplexes accumulated in the paper fiber pores (Figure 5b) and were partially visible to the naked eye. For quantitative analysis, the Raman peak intensities of 4-ATP at 1580 cm^−1^ were averaged from spectra collected at ten different points in the test line center. The SERS-based LFIA strip sensor demonstrated high sensitivity and quantitative analysis potential, promising swift and sensitive target antigen detection. In the future, with modifications in conjugation and antibody pairs, the SERS-LFIAS test could detect multiple pathogens. This quick, basic, and highly sensitive immunoassay is suitable for field testing AIV subtypes H7N9.

In another study, Maneeprakorn et al. [162] created a SERS-LFIA test system utilizing multi-branched gold nanostars (AuNS) coated with 4-aminothiophenol as a signal reporter for enhanced Raman scattering on a lateral flow immunochromatography platform. The distinct quality of AuNS with multiple branches and rough surface attributes enables the system to attain high SERS performance by enhancing bio-conjugation sites and hot spot regions. In order to show the performance of the system, influenza A nucleoprotein was utilized as the specific molecule. Following a simple one-step process, AuNS was labeled with the ATP molecule and linked to the antibody, particularly to influenza A nucleoprotein, serving as the SERS signal reporter and detection probe in the system. The study found that visual detection had a minimum detection limit of 67 ng mL^−1^, while SERS detection had a lower limit of 6.7 ng mL^−1^. The SERS detection signal significantly increased detection sensitivity by around 37 times compared to fluorescence-based tests, and by 300 times compared to traditional LFIA. The new SERS-LFIA platform showed an extremely high sensitivity in detection, paving the way for more accurate point-of-care testing in various illnesses.

**Figure 6:**
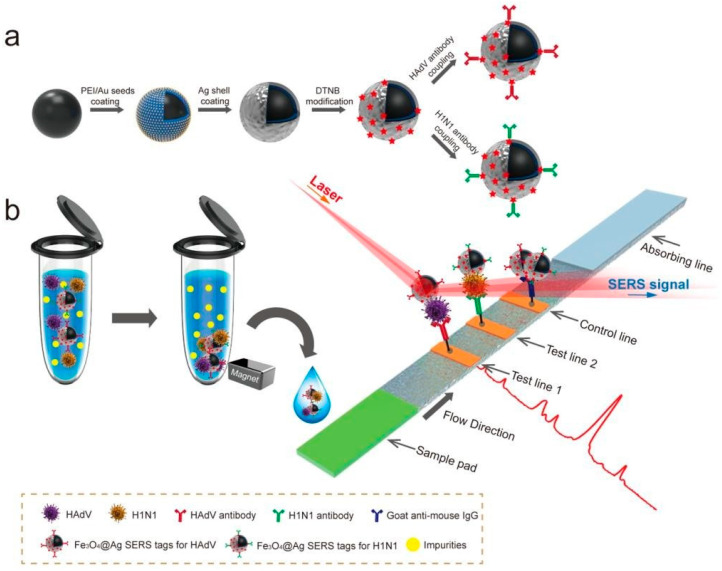
(**a**) Synthetic route for antibody-modified Fe_3_O_4_@Ag magnetic tags and (**b**) schematic diagram of magnetic SERS-strip for respiratory virus detection. Copyright/license 6100680611908; reproduced with permission from [177].

Another study by Wang et al. [177] highlighted the critical importance of rapid respiratory virus detection in preventing infection spread and directing proper treatment. In their study, they created a precise and quantitative lateral flow immunoassay (LFIA) strip with SERS technology to detect both influenza A H1N1 virus and human adenovirus (HAdV). This was achieved by utilizing Fe_3_O_4_@Ag nanoparticles as magnetic SERS nanotags. The new Fe_3_O_4_@Ag magnetic tags have dual-layer Raman dye molecules and virus-capture antibodies, and can specifically recognize and enrich target viruses in solution, as well as detect them on the strip using SERS (Figure 6). Using this approach, the magnetic SERS strip can be utilized with actual biological samples without the need for any preliminary sample preparation steps. The detection limits for H1N1 and HAdV were 50 and 10 pfu/mL, respectively, making them 2000 times more sensitive than the standard colloidal gold strip method. Additionally, the suggested strip is user-friendly, quick, steady, and capable of high throughput, making it a promising instrument for detecting virus infection early on.

**Figure 7:**
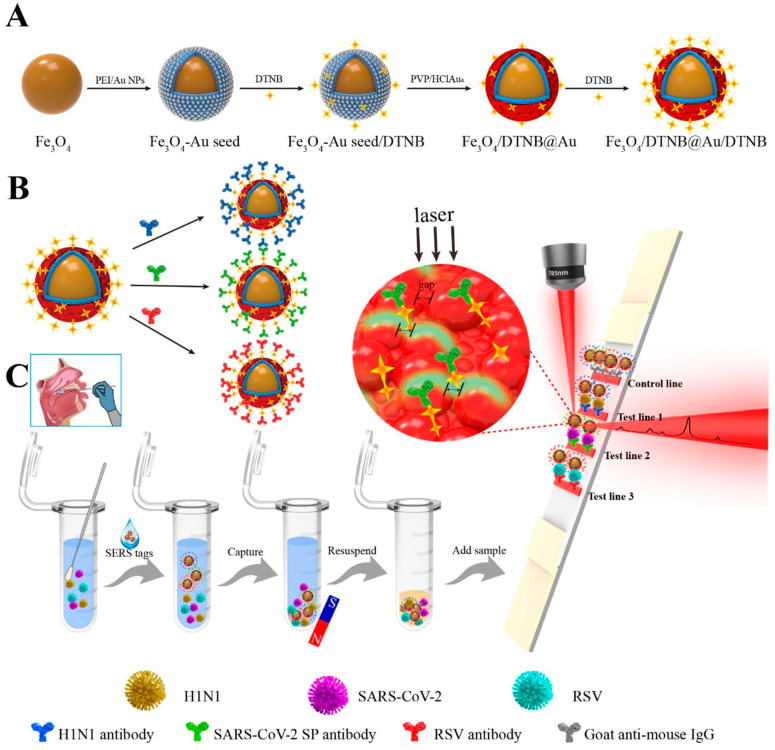
Quantitative detection of respiratory viruses via multichannel magnetic SERS-based LFA strip. (**A**) Synthesis of dual-layer DTNB-modified Fe_3_O_4_@Au MNPs. (**B**) Preparation of antibody-conjugated SERS tags for different respiratory viruses. (**C**) Collection of throat swab sample and operating procedure for the simultaneous quantitative detection of three respiratory viruses through the Fe_3_O_4_@Au-based SERS LFA strip. Copyright/license 6100560543267. Reproduced with permission from [4].

In a study, Liu et al. [4] presented a magnetic SERS-based lateral flow immunoassay with multiple channels for the sensitive and simultaneous detection of respiratory viruses, such as H1N1, SARS-CoV-2, and RSV. Fe_3_O_4_@Au MNPs (220 nm) with excellent monodispersity and powerful SERS enhancement were synthesized for high performance. Additionally, the presence of two layers of DTNB attached to the large core of Fe_3_O_4_@Au MNPs resulted in significant SERS signals and numerous conjugated sites for targeted antibodies. The Fe_3_O_4_@Au nanotags, prepared with three types of virus-specific capture antibodies, selectively trapped H1N1/SARS-CoV-2/RSV viruses from throat swab samples, eliminating impurities. The quantification of these viruses was achieved by analyzing SERS signals on the respective T lines (Figure 7). The detection limits of the new Fe_3_O_4_@Au-based SERS strips were 85 copies mL^−1^, 8 pg mL^−1^, and 8 pg mL^−1^ for H1N1, SARS-CoV-2, and RSV. This shows that our method’s sensitivity was enhanced 100 and 5–500 times compared to colloidal Au NP-LFA and ELISA. In addition, they demonstrated excellent accuracy and consistency in identifying actual respiratory viruses in throat swab samples with this method. According to the literature, this was the first attempt to develop a multichannel SERS-based LFA for detecting three respiratory viruses at the same time. Therefore, the suggested approach showed great promise as a diagnostic tool for quickly and accurately identifying respiratory viruses in field settings.

### 5.2. SERS-Antibody Probes for the Sensitive AIV Detection

A straightforward and extremely sensitive method for identifying the influenza virus using SERS antibody probes was developed by Moon et al. [178]. The preparation of SERS antibody probes is a straightforward process that involves combining gold nanoparticles with a gold-binding peptide-protein G and antibodies, thereby eliminating the need for complex chemical or biological reactions. Moreover, they ensure optimal conformation for the antibody’s attachment to Influenza A/CA/07/2009 (pH1N1), enabling precise detection of pH1N1. This method provides excellent specificity for the pH1N1 virus and can detect as little as 4.1 × 10^3^ TCID per mL. This approach can be used to detect hazardous substances and pathogens.

Pang et al. [179] have exposed a possible diagnostic tool for quickly detecting the HPAIV at the point of care. Utilizing the molecular sentinel probes adapted SERS surface allows for the identification of RNA target sequences associated with the N66S gene mutation in PB1-F2 protein linked to the HPAI virus, with a linear range from 0 to 60 attomoles and a detection limit of 2.67 attomoles. Future research will develop more MS probes for virus RNA markers, including HPAI. The probes will shift from DNA to locked nucleic acid or peptide nucleic acids for better RNA binding. A new SERS active substrate will be studied to increase sensitivity and reduce patient cell samples for diagnosis. Since this ultrasensitive biosensor assay does not require PCR amplification, it could serve as a potential diagnostic tool for detecting the HPAIV at the point of care.

### 5.3. SERS-Aptasensors for the Detection of Influenza Viruses

A new aptasensor that employs SERS for detecting various types of influenza viruses was developed by Kukushkin et al. [138]. The limit of detection was 10^4^ virus particles per sample or 10^−4^ HAU per sample, much lower than the values seen in other frequently used quick tests for detecting IV. The RHA0385 aptamer can be used to easily identify H1, H3, and H5 influenza virus subtypes. The analysis can be completed in just 12 min using affordable reagents, making it an attractive option for future use. Hence, aptasensors could be utilized for rapid and inexpensive strain-independent detection of influenza viruses.

Chen et al. [146] created an aptasensor using SERS for detecting influenza A/H1N1. The utilization of aptamers and nanomaterial nano-popcorn enhanced the sensitivity of the aptasensor and enabled a secure quantification of the virus target. The sensor showed a limit of detection of 97 PFU mL^−1^. It is around three times more sensitive than the ELISA, with a detection time of 20 min, proving to be an extremely sensitive and dependable tool for identifying viral pathogens. Surface energy differences between the per-fluorodecanethiol spacer and Au layer led to uniform self-assembly of Au nanoparticles, creating hotspots on the substrate for boosted incident field from localized surface plasmon effects. Multiple hotspots on the nano-popcorn substrate ensured reliable analysis of target molecules. Raman signal decreased with aptamer DNA binding A/H1N1 virus on the substrate, allowing accurate quantitative assessment of virus presence.

Gribanyov et al. [143] developed a novel method that combines quick, accurate detection with the ability to measure the number of viruses, as demonstrated in their study on influenza A. Aptamers imparted specificity to influenza hemagglutinin, while a SERS-based technique imparted sensitivity. The suggested method can be categorized as a quick diagnostic test because of its brief analysis time (less than 15 min) and straightforward sample preparation (the test is uniform). AgNPs were selected for their uncomplicated preparation and strong stability. Both absolute and relative SERS signal intensities are viable as analytical signals. The best aptasensor achieved a LOD of 2 × 10^5^ VP/mL and a detection range of 2 × 10^5^–2 × 10^6^ VP/mL. There was a need for rapid virus detection methods, with SERS offering high sensitivity. DNA aptamers can provide specificity for SERS biosensors. Existing aptasensors for virus detection have limitations, including a lack of quantification and complexity. A new method combining targeted identification and virus level measurement, exemplified by the influenza A virus, has been introduced to address these issues effectively.

### 5.4. SERS-Based Immunoassay Platform for the Detection of Influenza Viruses

A SERS immunoassay for influenza A that is both highly sensitive and selective, utilizing PEGylated TBBT-labeled AuNPs as probes and hydrophilic Au@Ag 2D array as substrates, was demonstrated by Karn-orachai et al. [66]. The unique Raman signal of TBBT (Raman reporter) allowed for the identification of the antibody–antigen interaction. Immunoassay on SERS substrates shows about four times higher sensitivity than immunoassay on Au film substrates, due to the enhancement of the Raman signal from SERS probes by the electromagnetic field effect of the SERS substrate. This immunoassay can detect the target nucleoprotein in a complex biological sample with various interferences at a detection limit of 6 TCID_50_ per mL. These results indicate that using a carefully tuned Au@Ag 2D array as a substrate for SERS can boost the sensitivity of SERS biosensors. It shows that this SERS immunosensor platform has the potential to detect target molecules with high sensitivity and specificity in complex clinical specimens.

Wang et al. [180] proposed utilizing a SERS-based immunoassay with Digital Microfluidics (DMF) for rapid, automated, and precise detection of disease biomarkers. SERS tags were designed using a core@shell nanostructure and were tagged with the Raman reporter 4-mercaptobenzoic acid (4-MBA), demonstrating strong signals, consistency, and durability. A sandwich immunoassay was developed by using magnetic beads coated with antibodies as a stable platform to capture antigens from samples, forming a bead-antibody–antigen immunocomplex. By utilizing a SERS tag that is functionalized with a detection antibody, the immunocomplex can be marked for sensitive detection of the antigen using the powerful SERS signal. The ability of DMF to automate tasks can make the assay process easier and decrease the chance of coming into contact with dangerous samples. The DMF-SERS method was used to showcase the usefulness of detecting AIV H5N1 in both buffer and human serum. The DMF-SERS technique offers exceptional sensitivity (detection limit of 4 pg/mL) and specificity for identifying H5N1, requiring less than an hour for the assay and using approximately 30 μL of reagents, which is less than the traditional ELISA method. Hence, the DMF-SERS technique shows promise for the precise and automatic identification of numerous infectious pathogens.

### 5.5. SERS-Immunomagnetic-Based AIV Detection

**Figure 8:**
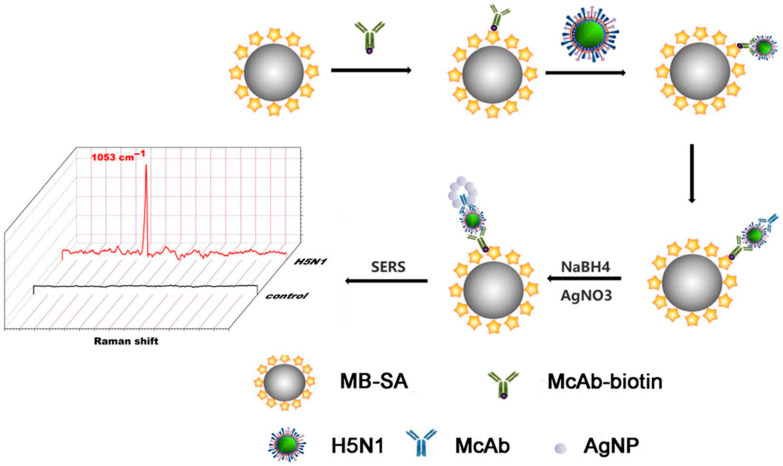
H5N1 influenza virus detection by IMBSIs@Ag-SERS method. Copyright/license https://creativecommons.org/licenses/by/4.0/; accessed on 1 September 2025, reproduced with permission from [181].

Wang et al. [181] used a SERS-based immunomagnetic bead to detect AIV, achieving a detection limit of 5.0 × 10^−6^ TCID_50_ per mL. Researchers developed a sandwich immunomagnetic bead SERS assay for the rapid detection of the H5N1 influenza virus with excellent specificity. The H5N1 influenza virus demonstrated its ability to attach to a biotinylated primary antibody on magnetic beads and was subsequently mixed with a secondary antibody to create immunomagnetic bead sandwich immunocomplexes (IMBSIs). The intense SERS signal from the H5N1 influenza virus can be effectively detected via the in situ reduction in nano-silver acting as a SERS substrate (Figure 8). No cross-reactivity with H1N1, H5N6, or H9N2 AI viruses was observed. The technique accurately detected H5N1 in chicken embryos, suggesting potential for novel influenza diagnosis using SERS. The key factor for using SERS in virus detection will be a method with improved capability, reliability, precision, and sensitivity. In the future, this Label-Free SERS method has the potential to be widely used for portable and quick Raman detection of many pathogens.

**Figure 9:**
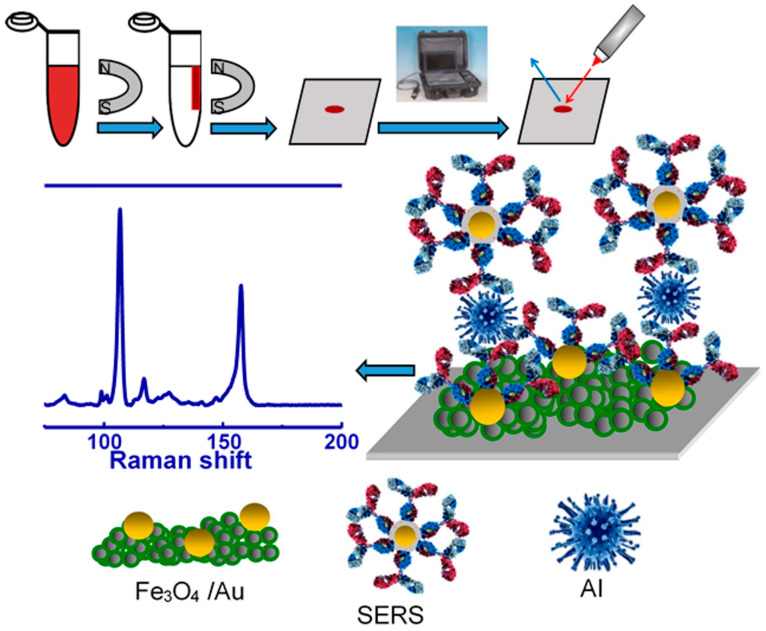
Representation of the SERS-based magnetic immunoassay. Copyright/license 6100560317913; reproduced with permission from [49].

Sun et al. [49] provided a novel approach for detecting AIV by utilizing 4-MBA-labeled AuNPs as SERS markers and Fe_3_O_4_/AuNPs with high SERS activity as substrates for support and capture. Figure 9 illustrates the SERS-based magnetic immunoassay protocol, which constructs a sandwich structure through specific antibody–antigen (virus) interactions. Magnetic substrates enable the concentration and isolation of viruses from complex samples, simplifying pretreatment. 4-mercaptobenzoic acid (4-MBA), an aromatic molecule with carboxyl and thiol groups, spontaneously chemisorbs onto Au nanoparticles via Au-S bonds, forming a stable self-assembled monolayer that reduces detachment and enhances experimental reproducibility. Activated with N (3-dimethylaminopropyl)-N′-ethylcarbodiimide hydrochloride (EDC)/NHS (N-hydroxysuccinimide), 4-MBA acts as a coupling agent to covalently attach Influenza A IgG and serves as an effective Raman reporter due to its strong Raman scattering. This enables the detection of distinctive SERS signals that specifically recognize and quantify influenza viruses. Unlike thioglycolic or α-lipoic acid, using 4-MBA simplifies SERS tag preparation and increases Raman reporter surface concentration, thereby improving the reproducibility and sensitivity of SERS analysis. This strategy enables an easy and precise method for identifying influenza viruses. The magnetic immunosensor displayed great sensitivity with a minimum detection concentration of 10^2^ to 5 × 10^3^ TCID_50_/mL for H3N2. Considering its sensitivity, portability, quick testing time, minimal sample volume requirement, and straightforward sample preparation process, the immunoassay created in this study is deemed suitable for practical use. A portable immunoassay with fast test time and simple preparation could be used to monitor AIV levels in human biological samples for quick diagnosis using a portable Raman spectrometer.

In another report, Chen et al. [182] developed a dual-mode surface-enhanced Raman scattering (SERS) aptasensor for the simultaneous and quantitative diagnosis of SARS-CoV-2 and Influenza A/H1N1. This platform employs a Au nanopopcorn substrate co-immobilized with specific DNA aptamers for each virus, tagged with distinct Raman reporters (Cy3 and RRX). An internal standard (4-MBA) was incorporated to normalize signal fluctuations and to normalize against instrumental and environmental variations. The intensity of the 1075 cm^−1^ peak from 4-mercaptobenzoic acid (4-MBA) was used as an internal standard. The detection mechanism is based on a signal-off strategy, where viral binding induces the detachment of the corresponding aptamer from the SERS substrate, resulting in a quantifiable decrease in signal intensity. This design enables the highly sensitive and specific discrimination and quantification of both pathogens in a single test, without cross-reactivity, thereby providing a novel platform for the rapid differentiation of respiratory infections to control the disease transmission.

### 5.6. SERS Applications for Diverse Pathogen Detection

The diagnostic potential of SERS is further demonstrated by its successful applications across diverse fields. For example, Abuhelwa et al. [183] developed a fiber-optic SERS sensor capable of directly detecting *Salmonella* in chicken rinsates within 10 min, thereby eliminating the requirement for a pre-enrichment step. The sensor operates by amplifying the Raman signal of cells captured near its hotspots, achieving a low LOD of 0.4–0.5 cells/mL and providing a foundation for rapid, evidence-based food safety measures. The developed fiber-optic SERS sensor represents a significant advancement in pathogen detection, demonstrating high specificity and sensitivity for *Salmonella* and *E. coli* O157:H7. Its capacity for rapid analysis is promised to revolutionize food safety standards, offering the poultry industry a vital tool for prompt intervention and significantly strengthened safety protocols.

Muthukumar et al. [184] reported a SERS immunosensor based on a silver-coated nanoporous silicon substrate, demonstrating its application for the rapid and sensitive detection of *E. coli* contamination in milk, which concludes that the miniaturized SERS platform is a reliable, rapid, and accurate tool for analyzing complex media, with strong potential for the routine on-site detection of emerging pathogens relevant to disease management. Wang et al. [185] reported a lateral flow immunoassay integrated with surface-enhanced Raman scattering (SERS-ICA) utilizing WS_2_-Au nanocomposites as SERS tags, enabling the ultrasensitive and quantitative detection of the foodborne pathogen *E. coli* O157:H7 in complex food samples. The proposed SERS assay enables the rapid, qualitative, and quantitative detection of *E. coli* O157:H7, demonstrating significant potential for field-based analysis.

Zavyalova et al. [186] developed a direct, one-step assembly protocol with colloidal nanoparticles to enable rapid SARS-CoV-2 detection within 7 min. The method achieves high sensitivity, with a limit of detection (LOD) of 5.5 × 10^4^ TCID50/mL, and high specificity. Several studies have demonstrated the application of SERS for monitoring and identifying pathogen contamination in various food samples. Recent literature contains multiple comprehensive studies focusing on the application of SERS technology for SARS-CoV-2 detection [187,188,189,190]. A recent review by Chen et al. [191] comprehensively examines the development of non-noble metal SERS substrates and their applications in analytical fields such as biomarker detection and environmental monitoring.

SERS has emerged as a front-line diagnostic tool by overcoming key limitations of traditional methods, such as sensitivity, speed, and multiplexing. Its foundation in plasmonic signal enhancement on nanoscale metallic surfaces provides a direct mechanism to solve persistent diagnostic challenges. The enormous enhancement factor of SERS (10^8^–10^11^) enables single-molecule detection, permitting the identification of trace biomarkers like cancer-derived exosomes or proteins at concentrations thousands of times lower than the detection threshold of conventional assays. The narrow spectral features of Raman reporters (<2 nm width) permit the simultaneous use of multiple unique SERS nanotags in a single assay. This facilitates parallel detection of biomarker panels from a minimal sample volume, yielding a comprehensive diagnostic profile for accurate disease stratification. SERS assays are now being integrated into user-friendly, rapid formats such as LFIAs and microfluidic chips. Consequently, the in situ label-free SERS method is a promising platform for the future of portable, rapid diagnostics, poised to enhance the practical deployment of Raman spectroscopy for infectious disease monitoring in clinical samples.

## 6. SERS-AIV Detection Challenges in Chickens and Future Prospects

Asymptomatic carriers (AIV-affected chickens with no signs of illness) play a critical role in disease dynamics. These birds silently transmit and maintain the virus in poultry populations, potentially leading to disease transmission to other birds or across different regions, making control efforts significantly more difficult [192].

A major challenge in controlling Avian Influenza is the virus’s broad host specificity, which facilitates transmission among diverse bird species and thereby complicates strain surveillance and transmission tracking in poultry. The virus exhibits cross-species transmission capability, potentially infecting mammals including humans. Swine serve as particularly concerning mixing vessels where viral reassortment can generate novel strains. This zoonotic potential represents a significant public health threat due to the severe disease manifestations in human cases. Individuals with occupational exposure, particularly poultry farm workers and those handling infected birds or contaminated environments, face elevated infection risks, highlighting the critical intersection of animal and human health in AI management [172,193]. AI in chickens leads to rapid genetic changes, raising concerns about the development of new strains with different characteristics, like increased strength, vaccine resistance, and cross-species infection potential. Over time, the virus undergoes gradual genetic variations, particularly in surface protein genes like HA and NA, crucial for invading host cells and evading the immune system. These changes may result in the creation of novel virus strains evading immune detection through antigenic drift, or the sudden emergence of a new variant via reassortment, posing a potential pandemic threat [171,194].

Chickens are highly susceptible to co-infections with multiple pathogens, including various AIV strains. These concurrent infections complicate accurate AIV diagnosis, increasing the risk of false-negative results. Furthermore, they pose a significant threat by facilitating genetic reassortment, which can generate novel, more virulent, and transmissible viral strains [195,196]. The collection and processing of samples from AIV-infected poultry represents an essential but hazardous undertaking, requiring rigorous biosecurity measures to contain the highly contagious pathogen. This process demands careful handling of infected birds and strict maintenance of sample integrity to prevent cross-contamination while preserving diagnostic reliability. Successful implementation of these protocols ensures accurate test results that form the foundation of effective outbreak containment strategies and informed disease management decisions [197,198]. The ability of diagnostic tests to accurately detect the AIV in chickens is known as the sensitivity of detection methods (even at low levels). High diagnostic sensitivity is essential for the timely detection of the virus, effective outbreak control, and minimizing its spread between poultry populations. Traditional diagnostic methods may lead to false-positive or false-negative results [69,91].

Addressing these challenges requires the development and implementation of sensitive, rapid, and reliable detection methods tailored to the specific characteristics of the AIV in chickens. Advances in technology, such as SERS, offer promising solutions for improving the detection and monitoring of AI viruses in poultry populations. Despite the promising results, there are still several challenges and limitations associated with the use of SERS for virus detection. The performance of SERS substrates can vary depending on their composition, morphology, and preparation method, ensuring consistent and reproducible SERS substrates is crucial for reliable virus detection [142]. The use of SERS for detecting AIV in chickens faces significant challenges due to matrix effects in clinical samples. Naso-pharyngeal swabs and saliva contain various contaminants that can interfere with the SERS signal or lead to non-specific adsorption, requiring the development of specific sample preparation and data analysis protocols to mitigate these issues [199,200]. The establishment of these protocols is vital for the clinical implementation of SERS [201].

The current literature on SERS-based detection relies predominantly on model or spiked samples. consequently, a critical next step is rigorous validation with clinical specimens to assess its true diagnostic capability. The validation process must include an assessment of reproducibility, which involves conducting multiple investigations on the same sample and repeating experiments across different groups to evaluate consistency. Furthermore, the validation of the SERS technique must be evaluated by determining its sensitivity and specificity. These metrics, which define the capacity to recognize low analyte concentrations and the capability to differentiate the target analyte from other substances, should be compared against gold standard clinical methods such as ELISA and PCR, etc. [202].

The future integration of SERS into AIV surveillance is highly promising, depending on two key factors: first, the development of novel substrates with enhanced sensitivity, specificity, and reproducibility; and second, their integration into microfluidic systems paired with portable spectrometers to provide practical point-of-care investigations for rapid AIV detection. Moreover, a comprehensive biosecurity strategy is crucial for preventing and controlling AI in poultry. An effective strategy must integrate strict farm management, environmental controls, and bird management, supported by a robust emergency plan. Although implementation of these protocols presents significant challenges, strict implementation of these protocols is necessary to mitigate outbreak risks, maintain flock health, and protect public health.

## 7. Concluding Remarks

In conclusion, SERS demonstrates exceptional theoretical potential for revolutionizing poultry health monitoring; several critical research gaps must be addressed to realize its practical implementation. The core strengths of SERS, including its single-molecule sensitivity, rapid detection speed, and inherent multiplexing capacity, offer compelling advantages over conventional methods for AIV surveillance. However, the field currently faces significant challenges in substrate reproducibility, with most studies relying on idealized laboratory conditions rather than validated field applications. The predominant use of spiked samples rather than clinical specimens from naturally infected birds represents a significant obstacle to accurately assessing the diagnostic potential for poultry health monitoring.

Furthermore, the successful integration of SERS platforms into automated farm systems requires substantial development in sample processing automation, data interpretation procedures, and operational robustness for untrained operators. The realistic potential of SERS lies not in immediately replacing established techniques like RT-PCR, but in creating complementary rapid-screening networks that operate at the point-of-need. Future research should prioritize the development of cost-effective, stable substrates, validate assays across diverse poultry species and sample matrices, and establish standardized protocols for reliable quantification. As these technical and translational challenges are systematically addressed, SERS is positioned to emerge as a keystone technology in smart poultry farming, enabling real-time pathogen surveillance, comprehensive flock health assessment, and ultimately contributing to more resilient global food security systems through data-driven biosecurity management. Moreover, SERS can also be modified to detect other viruses, bacteria, and even environmental toxins, making it a flexible tool in both medical and environmental fields.

## Data Availability

No new data were created or analyzed in this study. Data sharing is not applicable to this article.

## Supplementary Materials

The following supporting information can be downloaded at: https://www.mdpi.com/article/10.3390/vetsci12111052/s1, Table S1: Key Advantages/Disadvantages and Comparative Analysis: SERS Vs. Traditional Methods for AIV Detection; Table S2: SERS applications for highly sensitive AIV detection.
