# Supplementary material for: Overcoming Challenges in Avian Influenza Diagnosis: The Role of Surface-Enhanced Raman Spectroscopy in Poultry Health Monitoring
Source: Vet Sci. 2025 Nov 2;12(11):1052. doi: 10.3390/vetsci12111052 (PMC12656975; doi:10.3390/vetsci12111052)
Supplement: Supplementary file 1 [file vetsci-12-01052-s001.zip › vetsci-3880549-supplementary.pdf]

Supplementary Information

Overcoming Challenges in Avian Influenza Diagnosis: The Role of Surface-Enhanced Raman Spectroscopy in Poultry Health Monitoring

Muhammad Farhan Qadir [1,2,*] and Yukun Yang [2]

[1] College of Life Sciences, Henan Normal University, Xinxiang 453007, China
[2] School of Life Science, Shanxi University, Taiyuan 030006, China; yangyukun@sxu.edu.cn
* Correspondence: farhanqadirvet@gmail.com or drfarhanqadir@gmail.com

Supplementary Table S1: Key Advantages/Disadvantages and Comparative Analysis: SERS Vs. Traditional Methods for AIV Detection

Feature	SERS	RT-qPCR	ELISA	LAMP	Rapid antigen tests	Virus Isolation
**Principle**	Enhances the Raman signal of molecules on nanostructured metal surfaces	Amplifies and detects specific RNA sequences using fluorescent probes during thermal cycling	Detects viral antigens/antibodies using enzyme-linked antibodies and a colorimetric reaction	Isothermal nucleic acid amplification using 4-6 primers at constant temperature (60-65°C)	Detects the presence of viral antigens (proteins) using specific antibodies on a lateral flow strip	Grows and amplifies infectious virus particles in a susceptible biological system (e.g., embryonated chicken eggs or cell culture)
**Sensitivity**	Ultra-high; Can detect single virions or low copies	High: Gold standard, detects few RNA copies	Moderate: Often fails in early infection/low viral shedding	High: Comparable to PCR, detects 10-100 copies	Poor sensitivity (High false-negative outcomes)	High
**Specificity**	High: Structural fingerprinting of the entire virion	Very High: Sequence-specific primer/probe binding	Moderate: Antibody cross-reactivity possible	High: primers increase specificity but risk non-specific amplification	Moderate to high	Gold standard specificity (Conclusive identification)
**Multiplexing**	Excellent: Simultaneous detection of multiple subtypes	Moderate: Limited by fluorescent channels	Poor: Typically, single analyte detection	Difficult: (Technically challenging)	Very Poor (cannot distinguish between different viruses)	Cannot distinguish between different viruses/serotypes
**Speed**	10-30 minutes	1.5-4 hours	2-4 hours	30-90 minutes	<15 minutes	3-7 days
**Portability**	High: Handheld spectrometers enable true POC use	Low: Requires sophisticated thermocyclers	Moderate	Moderate	No equipment needed	BSL-3 facilities needed
**Commercial Availability**	Limited	Well-established kits	Commercial kits available	Increasing commercial options	Available	Not available / classical laboratory technique
**Cost**	High initial equipment cost	High equipment and reagent cost	Low cost per test	Moderate cost	Low cost	Expensive and labor-intensive
**References**	[4,162,176-179,181]	[39,92,93, 150]	[40, 41, 150]	[94-96,98]	[150]	[37,38,11,70]

Data from Liu et al. [4], [Nanomedicine: Nanotechnology, Biology, and Medicine]; published by [Elsevier], [2023]. Maneeprakorn et al. [162], [RSC Advances]; published by [The Royal Society of Chemistry], [2016]. Xiao et al. [176], [Analytica Chimica Acta]; published by [Elsevier], [2019]. Wang et al. [177], [Applied Materials & Interfaces]; published by [American Chemical Society], [2019]. Moon et al. [178], [RSC Advances]; published by [The Royal Society of Chemistry], [2016]. Pang et al. [179], [Biosensors and Bioelectronics]; published by [Elsevier], [2014]. Wang et al. [181], [Influenza and Other Respiratory Viruses]; published by [John Wiley and Sons], [2023]. Augustine et al. [39], [Biology (Basel)]; published by [MDPI], [2020]. Doak et al. [92], [Methods in Molecular Biology]; published by [Springer], [2012]. Chen et al. [93], [Journal of Medical Microbiology]; published by [Microbiology Society], [2007]. Lozano Gómez et al. [150], [Medicina Clínica] published by [Elsevier], [2020]. Vasilyeva et al. [40], [Sensors] published by [MDPI], [2025]. Liu et al. [41], [Biosensors and Bioelectronics] published by [Elsevier], [2021]. Notomi et al. [94], [Nucleic Acids Research]; published by [Oxford University Press], [2000]. Dinh et al. [95], [Tropical Medicine and Health]; published by [J-STAGE], [2011]. Imai et al. [96], [Vaccine]; published by [Elsevier], [2006]. Jung et al. [98], [Analytica Chimica Acta]; published by [Elsevier], [2015]. Hong et al. [37], [Angewandte Chemie, International Edition]; published by [Wiley], [2018]. Vemula et al. [38], [*Viruses*]; published by [MDPI], [2016]. Swayne et al. [11], [Immunological Reviews]; published by [Wiley], [2008]. Hoffmann et al. [70], [Archives of Virology]; published by [Springer], [2001].

Supplementary Table S2. SERS applications for highly sensitive AIV detection

Year	Country	Strain	Host	Detection target/transducer/ Nanomaterial	Limit of detection (LOD)	References
SERS-lateral flow immunoassay-based AI viral detection						
2019	China	H7N9 AIV	Chicken	SERS-based lateral flow immunoassay strip (LFIAS)	0.0018 HAU LOD for H7N9	[176]
2016	Thailand	Influenza A virus	Avian	SERS-based lateral flow immunoassay (LFIA)	LOD using visual and SERS detection was 67 and 6.7 ng mL^{-1}, respectively	[162]
2019	China	H1N1	Avian	SERS-based LFIA	LOD for H1N1 and HAdV were 50 and 10 pfu/mL, respectively	[177]
2023	China	H1N1	Avian	SERS-based LFA	8 pg mL^{-1} for H1N1	[4]
SERS-antibody probes for the sensitive AIV detection						
2016	Korea	H1N1	Avian	SERS antibody probes	4.1×10^3 TCID/mL	[178]
2014	China	HPAIV	Avian Birds	Au–Gr-FON substrate was the SERS-active substrate	LOD was 2.67 attomoles	[179]
SERS-aptasensors for the detection of influenza viruses						
2019	Russia	Various AIV strains	Avian	SERS-based Aptasensor for the detection of viruses	LOD was 10^{-4} HAU/sample	[138]
2020	China	H1N1	Chicken	SERS-aptasensor	97 PFU mL^{-1}	[146]
2021	Russia	Influenza A virus	Avian	SERS on colloidal AgNPs and specific recognition by aptamers	2×10^5 VP/mL with a dynamic range of 2×10^5–2×10^6 VP/mL	[143]
SERS-based immunoassay platform for the detection of influenza viruses						
2016	Japan	Influenza A virus	Avian	SERS probes and hydrophilic Au@Ag 2D array as the SERS substrates	6 $TCID_{50}$/mL	[66]
2018	China	H5N1	Avian	SERS-based immunoassay	4 pg/mL	[180]
SERS-immunomagnetic-based AIV detection						
2023	China	H5N1	Avian	SERS-based immunomagnetic bead	5.0×10^{-6} $TCID_{50}$/mL	[181]
2017	China	H3N2	Avian birds	4-MBA-labeled Au NPs as SERS tags with SERS-active Fe_3O_4/Au NPs magnetic immunosensor	10^2 to 5×10^3 $TCID_{50}$/mL	[49]

Data from Xiao et al. [176], [Analytica Chimica Acta]; published by [Elsevier], [2019]. Maneeprakorn et al. [162], [RSC Advances]; published by [The Royal Society of Chemistry], [2016]. Wang et al. [177], [Applied Materials & Interfaces]; published by [American Chemical Society], [2019]. Liu et al. [4], [Nanomedicine: Nanotechnology, Biology, and Medicine]; published by [Elsevier], [2023]. Moon et al. [178], [RSC Advances]; published by [The Royal Society of Chemistry], [2016]. Pang et al. [179], [Biosensors and Bioelectronics]; published by [Elsevier], [2014]. Kukushkin et al. [138], [PLoS ONE]; published by [Public Library of Science], [2019]. Chen et al. [146], [Biosensors and Bioelectronics]; published by [Elsevier], [2020]. Gribanyov et al. [143], [International Journal of Molecular Sciences]; published by [MDPI], [2021]. Karn-orachai et al. [66], [RSC Advances]; published by [The Royal Society of Chemistry], [2016]. Wang et al. [180], [Analytical Chemistry]; published by [American Chemical Society], [2018]. Wang et al. [181], [Influenza and Other Respiratory Viruses]; published by [John Wiley and Sons], [2023]. Sun et al. [49], [Biosensors and Bioelectronics]; published by [Elsevier], [2017].
